# Risk factors associated with prolonged intensive care unit stay following surgery for total anomalous pulmonary venous connection: a retrospective study

**DOI:** 10.1186/s13019-023-02356-5

**Published:** 2023-09-09

**Authors:** Jinjin Huang, Jian Tang, Yong Fan, Dongpi Wang, Lifen Ye

**Affiliations:** 1 Department of Anesthesiology, Children’s Hospital, Zhejiang University School of Medicine, National Clinical Research Center for Child Health, Hangzhou, China; 2 Department of Extracorporeal Life Support, Heart Institute, Children’s Hospital, Zhejiang University School of Medicine, National Clinical Research Center for Child Health, Hangzhou, China

**Keywords:** Total anomalous pulmonary venous connection, Congenital heart disease, Neonate, Cardiovascular abnormalities, Mechanical ventilation, Intensive care unit, Length of stay, Critical illness

## Abstract

**Background:**

Prolonged intensive care unit (ICU) stays consume medical resources and increase medical costs. This study identified risk factors associated with prolonged postoperative intensive care unit (ICU) stay in children with total anomalous pulmonary venous connection (TAPVC).

**Methods:**

The medical records of 85 patients who underwent surgical repair of TAPVC were retrospectively analyzed. The patients were divided into prolonged-stay and standard-stay groups. The prolonged stay group included all patients who exceeded the 75th percentile of the ICU stay duration, and the standard stay group included all remaining patients. The effects of patient variables on ICU stay duration were investigated using univariate and logistic regression analyses.

**Results:**

Patient median age was 41 (18–103) days, and median weight was 3.80 (3.30–5.35) kg.Postoperative duration of ICU stay was 11–68 days in the prolonged stay group (n = 23) and 2–10 days in the standard stay group (n = 62). Lower preoperative pulse oximetry saturation (SpO_2_), higher intraoperative plasma lactate levels, and prolonged postoperative mechanical ventilation were independent risk factors for prolonged ICU stay. Preoperative SpO_2_ < 88.5%, highest plasma lactate value > 4.15 mmol/L, and postoperative mechanical ventilation duration was longer than 53.5 h, were associated with increased risk of prolonged ICU stay. Young age, low body weight, subcardiac type, need for vasoactive drug support, emergency surgery, long anesthesia time, low SpO_2_ after anesthesia induction, long cardiopulmonary bypass (CPB) and aortic clamp times, high lactate level, low temperature, large volume of ultrafiltration during CPB, large amounts of chest drainage, large red blood cells (RBCs) and plasma transfusion, and postoperative cardiac dysfunction may be associated with prolonged ICU stay.

**Conclusions:**

Lower preoperative SpO_2_, higher intraoperative plasma lactate levels, and prolonged postoperative mechanical ventilation were independent risk factors for prolonged ICU stay in children with TAPVC. When SpO_2_ was lower than 88.5%, the highest plasma lactate value was more than 4.15 mmol/L, and the postoperative mechanical ventilator duration was longer than 53.5 h, the risk of prolonged ICU stay increased. Improved clinical management, including early diagnosis and timely surgical intervention to reduce hypoxia time and protect intraoperative cardiac function, may reduce ICU stay time.

## Introduction

The incidence of total anomalous pulmonary venous connection (TAPVC) is 0.008% in live births, but it occurs in 2–3% of cases of congenital heart disease (CHD) [1–3]. In recent years, the incidence of complex CHD has declined with the promotion of prenatal screening. However, TAPVC remains a critical cardiac disease with the low prenatal detection rates. Routine ultrasound screening in mid-gestation detects TAPVC in only 2–10% of cases [4]. The outcomes of surgical repair of TAPVC have improved over the years. Critical care is an important stage in postoperative recovery. A prolonged intensive care unit (ICU) stay consumes medical resources and increases medical costs. Newburger et al. suggested that a prolonged postoperative ICU stay is independently associated with impaired cognitive function in children aged 8 years old [5]. Our previous study demonstrated that increased post-bypass serum lactate levels, the need for a larger volume of resuscitation fluid on postoperative day 1 (POD 1), and noninfectious pulmonary complications were independent risk factors for prolonged recovery in the surgical intensive care unit (SICU) after an arterial switch operation [6]. In this study, we analyzed the medical records of patients who underwent TAPVC surgery in the past five years to identify the risk factors associated with prolonged ICU stay to further improve clinical outcomes, decrease medical costs, and save medical resources.

## Materials and methods

### Patients

The medical records of patients who underwent surgical repair of TAPVC at our hospital between June 2016 and September 2020 were retrospectively analyzed. Patients who died after surgery couldn’t be assessed the length of ICU stay and were excluded from this study.

This study was approved by the Ethics Committee of the Children’s Hospital, Zhejiang University School of Medicine (approval number:2021-IRB-153).

### Clinical data collection

The following data were collected: demographic data; TAPVC anatomical subtypes; and preoperative, intraoperative, and postoperative variables. Patient demographic data included age, body weight, and sex. The preoperative variables included preoperative blood gas and lactate levels, whether the procedure was performed as an emergency, and vasoactive index score (VIS). Intraoperative variables included anesthesia time (from anesthesia induction to surgery), mean arterial pressure (MAP), pulse oxygen saturation (SPO_2_) after anesthesia induction, cardiopulmonary bypass (CPB) time, aortic clamp time, MAP during CPB, lowest body temperature, lowest hematocrit (HCT), highest plasma lactate level, ultrafiltrate volume, and fluid balance during CPB. The postoperative variables included blood gas, lactate level, cardiac function index (interventricular septum (IVS), MAP, creatine kinase MB isoenzyme (CK-MB)), arrhythmia, postoperative mechanical ventilator duration, pulmonary complications, and liver and kidney function.

### Surgical strategy

Patients who presented with severe respiratory distress and metabolic acidosis requiring intubation and inotropic support underwent emergency surgery. If the inner milieu was assessed abnormal and the estimated risk of surgery was high, extracorporeal membrane oxygenation (ECMO) was initiated to optimize the metabolic milieu. The remaining patients underwent planned surgery. For surgical management, there have been no changes in the devices used, medical teams, and surgical techniques over the past five years. A combination of intravenous and inhalation anesthesia was administered to all patients. Central venous catheterization and continuous arterial blood pressure (ABP) monitoring were performed after induction. The surgical field was sterilized and routinely exposed. Systemic heparinization was implemented to obtain a satisfactory activated clotting time (ACT) before establishing CPB by cannulating the ascending aorta and the superior and inferior vena cava. Surgical repair of TAPVC and associated intracardiac structural abnormalities was performed under moderately hypothermic CPB. After completion of the correction, the body was rewarmed to achieve the return of the spontaneous circulation. Vasoactive drugs were administered to achieve satisfactory ABP and oxygenation, and then separated from CPB. Reoperation was required if separation from CPB failed as a result of anastomotic stenosis. ECMO was initiated to assist if the traditional treatment for left heart dysfunction or pulmonary hypertension was ineffective. The child was cared for postoperatively in the cardiac ICU.

### The definition of prolonged ICU stay

The patients were transferred to the ward when they met the criteria for ICU discharge. The criteria included comfortable breathing with supplemental oxygen administered via a nasal cannula at 1 l/min or less; a fraction of inspired oxygen of 0.3 or less; stable and normal hemodynamics, heart rate, and blood pressure; adequate tissue perfusion [7]. There is no clear definition of a prolonged ICU stay following cardiac surgery. We referred to the definition of Xiwang Liu et al. [6]. Accordingly, the patients in this study were classified into two groups according to ICU stay duration: the prolonged stay group and the standard stay group. The prolonged stay group included all patients with an ICU stay exceeding the 75th percentile, and the standard stay group included all the remaining patients[6].

### Statistical analysis

Descriptive data for continuous variables are presented as mean ± standard deviation or as median and range as appropriate. Descriptive statistics for categorical variables are presented as percentages or counts. Univariate analysis was performed to compare the demographic data and pre-, intra-, and postoperative variables of the prolonged stay patients with those of the standard stay patients. A *P* value lower than 0.05 was considered statistically significant. Comparisons between the two groups were performed using the unpaired Student’s t-test or the Mann–Whitney U test for continuous variables and the chi-square test for categorical variables. Logistic regression analysis was used to identify independent risk factors for prolonged stay. Variables with a *P* value lower than 0.2 in univariate analysis were enrolled in this regression model. Statistical analysis was performed using IBM Statistical Package for the Social Sciences (SPSS) version 23.0.

## Results

A total of 95 children with TAPVC underwent surgery during the study period. Ten children died after surgery were excluded. Five of them died in 24 h and the other five children died on 7, 13, 17, 19, 22 days after surgery respectively. All of them died for dynamic failure with low cardiac output and even five of them with sepsis. After excluding the 10 deaths, 85 children were included in this study. Of these 85 children, three patients received temporary ECMO support for difficult weaning from extracorporeal circulation. The age of all 85 children was 41 (18–103) days, weight was 3.80 (3.30–5.35) kg, and hospitalization time was 26.0 (19.5–33.5) days. The children in the standard stay group (62 cases) stayed in the ICU for 2–10 days after surgery, whereas those in the prolonged stay group (23 cases) stayed for 11–68 days.

Table 1 presents the results of the univariate analysis comparing the preoperative risk factors of children in the prolonged and standard stay groups. Children in the prolonged stay group were younger and heavier than those in the standard group. Among the risk factors, the use of vasoactive drugs and the proportion of emergency surgeries were significantly higher in the prolonged stay group than those in the standard group.

**Table 1:** Univariate analysis of demographic and preoperative characteristics

Variable	Standard stay group (n = 62)	Prolonged stay group (n = 23)	*P*
**Age (d)**	48.50 (28.25-114.75)	14.00 (10.00–59.00)	**0.006**
**Weight(kg)**	4.05 (3.40–5.43)	3.30 (2.90–4.70)	**0.036**
**Sex (male/%)**	34 (54.8%)	16 (69.6%)	0.220
**Preoperative pH**	7.394 ± 0.009	7.388 ± 0.012	0.713
**Preoperative lactate(mmol/L)**	2.05 (1.20–3.20)	2.20 (1.20–3.30)	0.866
**Vasoactive drugs (n/%)**	7 (11.3%)	9 (39.1%)	**0.009**
**Pneumonia (n/%)**	38 (61.3%)	16 (69.6%)	0.481
**Emergency surgery (n/%)**	6 (9.7%)	9 (39.1%)	**0.009**

Table 2 shows the results of the univariate analysis comparing the intraoperative risk factors of the children in the prolonged and standard stay groups. Among these risk factors, longer anesthesia time, lower SpO_2_, longer CPB time, longer aortic cross-clamp (ACC) time, lower nasopharyngeal temperature (NPT), higher lactate level in CPB, and greater ultrafiltration (UF) volume were observed in the prolonged stay group.

**Table 2:** Univariate analysis of intraoperative characteristics

Variable	Standard stay group (n = 62)	Prolonged stay group (n = 23)	*P*
**Duration of anesthesia (min)**	240.26 ± 7.23	277.13 ± 13.71	**0.013**
**MAP after induction (mmHg)**	52.84 ± 1.28	50.70 ± 1.66	0.361
**SpO**_**2**_ **after induction (%)**	92.0 (89.0–96.0)	88.0 (82.0–94.0)	**0.039**
**Duration of CPB (min)**	99.00 (78.75-123.25)	130.00 (112.00-170.00)	**0.000**
**Duration of ACC (min)**	67.55 ± 28.01	88.09 ± 26.42	**0.003**
**Lowest NPT in CPB (°)**	29.55 (26.70-31.13)	26.70 (24.10–29.00)	**0.001**
**Lowest HCT in CPB (%)**	26.14 ± 0.47	24.77 ± 0.75	**0.132**
**Highest level of lactate in CPB (mmol/L)**	4.30 (3.60–5.58)	5.70 (4.40–8.30)	**0.005**
**MAP in CPB (mmHg)**	46.50 (38.00-54.25)	46.00 (40.00–51.00)	0.797
**UF volume (ml/kg)**	91.88 (65.79-134.23)	142.86 (97.96-196.97)	**0.001**
**Fluid balance in CPB (ml/kg)**	10.84 ± 3.73	4.56 ± 9.59	0.546

Abbreviations: MAP, mean arterial pressure; SpO_2_, pulse oxygen saturation; ACC, aortic cross-clamp; NPT, nasopharyngeal temperature; HCT, hematocrit; UF, ultrafiltration

Table 3 shows the results of the univariate analysis comparing the postoperative risk factors of the children in the prolonged and standard stay groups. Among these risk factors, PaO_2,_ lactate and blood calcium levels on POD 1 had a *P* value less than 0.2. These factors were included in the multivariate analysis. Similarly, there were significant difference between the two groups in the volume of chest drainage over 24 h after surgery, RBC and fresh-frozen plasma (FFP) infusion, IVS, MAP after surgery, arrhythmias, duration of mechanical ventilation, liver function, and coagulation function. All these risk factors were included in multivariate analysis.

**Table 3:** Univariate analysis of postoperative hemodynamic and laboratory variables

Variable	Standard stay group (n = 62)	Prolonged stay group (n = 23)	*P*
**pH on POD 1**	7.360 ± 0.008	7.342 ± 0.015	0.255
**PaO**_**2**_ **on POD 1 (mmHg)**	183.2 ± 10.55	237.07 ± 19.75	**0.012**
**HB in POD 1 (g/L)**	110.14 ± 2.28	106.83 ± 4.36	0.471
**Lactate on POD 1 (mmol/L)**	2.70 (1.88–4.43)	4.10 (3.20–5.10)	**0.011**
**Blood calcium on POD 1 (mmol/L)**	1.33 (1.23–1.44)	1.18 (1.12–1.35)	**0.007**
**Volume of chest drainage over 24 h after surgery (ml/kg)**	12.98 (8.31–18.76)	17.86 (11.70-26.79)	**0.016**
**RBC infusion (u/kg)**	0.46 (0.29–0.67)	1.33 (0.76–1.47)	**< 0.001**
**FFP infusion (ml/kg)**	69.09 ± 4.13	142.18 ± 13.83	**< 0.001**
**IVS (mm)**			
**POD 1**	14.11 ± 1.26	22.21 ± 2.38	**0.002**
**POD 2**	10.35 ± 1.09	20.78 ± 2.66	**0.001**
**POD 3**	6.65 (0.58–11.75)	16.30 (12.40–24.70)	**< 0.001**
**LVEF(%)**			
**POD 1**	67.15 ± 1.85	65.60 ± 3.36	0.663
**POD 2**	67.72 ± 1.42	66.50 ± 5.98	0.854
**POD 3**	66.70 ± 2.77	68.57 ± 0.97	0.537
**CK-MB on POD 1 (U/L)**	75.00 (60.50–93.50)	81.50 (58.50-114.75)	0.549
**MAP(mmHg)**			
**24 h after surgery**	65.85 ± 1.08	63.70 ± 1.79	0.303
**48 h after surgery**	67.84 ± 0.85	62.13 ± 1.81	**0.002**
**72 h after surgery**	67.65 ± 0.90	65.17 ± 1.67	**0.171**
**Arrhythmias (n/%)**	46 (70.7%)	21 (91.3%)	**0.047**
**Duration of mechanical ventilation (hours)**	25 (22–47)	117(58–163)	**< 0.001**
**Pneumonia (n/%)**	52 (80.0%)	22 (95.6%)	0.283
**Pneumonic consolidation (n/%)**	6 (9.2%)	1 (4.3%)	0.726
**Pneumothorax (n/%)**	15 (23.1%)	7 (30.4%)	0.559
**Renal function**			
**Cystatin C (mg/L)**	1.22 (0.97–1.48)	1.25 (1.05–1.35)	0.847
**Peritoneal dialysis (n/%)**	2 (3.1%)	1 (4.3%)	1.000
**Liver function**			
**ALT (U/L)**	17.00 (12.75-23.00)	13.00 (8.00–19.00)	**0.130**
**TBIL (mmol/L)**	34.60 (18.93–91.20)	98.60 (35.00-129.10)	**0.011**
**DBIL (mmol/L)**	9.95 (5.95–12.40)	11.80 (9.50–16.10)	**0.021**
**Coagulation function**			
**PT (s)**	13.80 (12.48–16.13)	14.30 (13.00-16.60)	0.392
**APTT (s)**	39.30 (32.45–43.38)	50.80 (37.50–59.10)	**0.005**
**INR**	1.19 (1.08–1.36)	1.19 (1.06–1.36)	0.74

Abbreviations: pH, potential hydrogen; POD, postoperative day; PaO_2_, partial pressure of arterial oxygen; HB, hemoglobin; RBC, red blood cells; FFP, fresh-frozen plasma; IVS, interventricular septum; LVEF, left ventricular ejection fraction; CK-MB, creatine kinase MB isoenzyme; MAP, mean arterial pressure; ALT, alanine aminotransferase; TBIL, total bilirubin; DBIL, direct bilirubin; PT, prothrombin time; APTT, activated partial thromboplastin time; INR, international normalized ratio

**Fig. 1:**
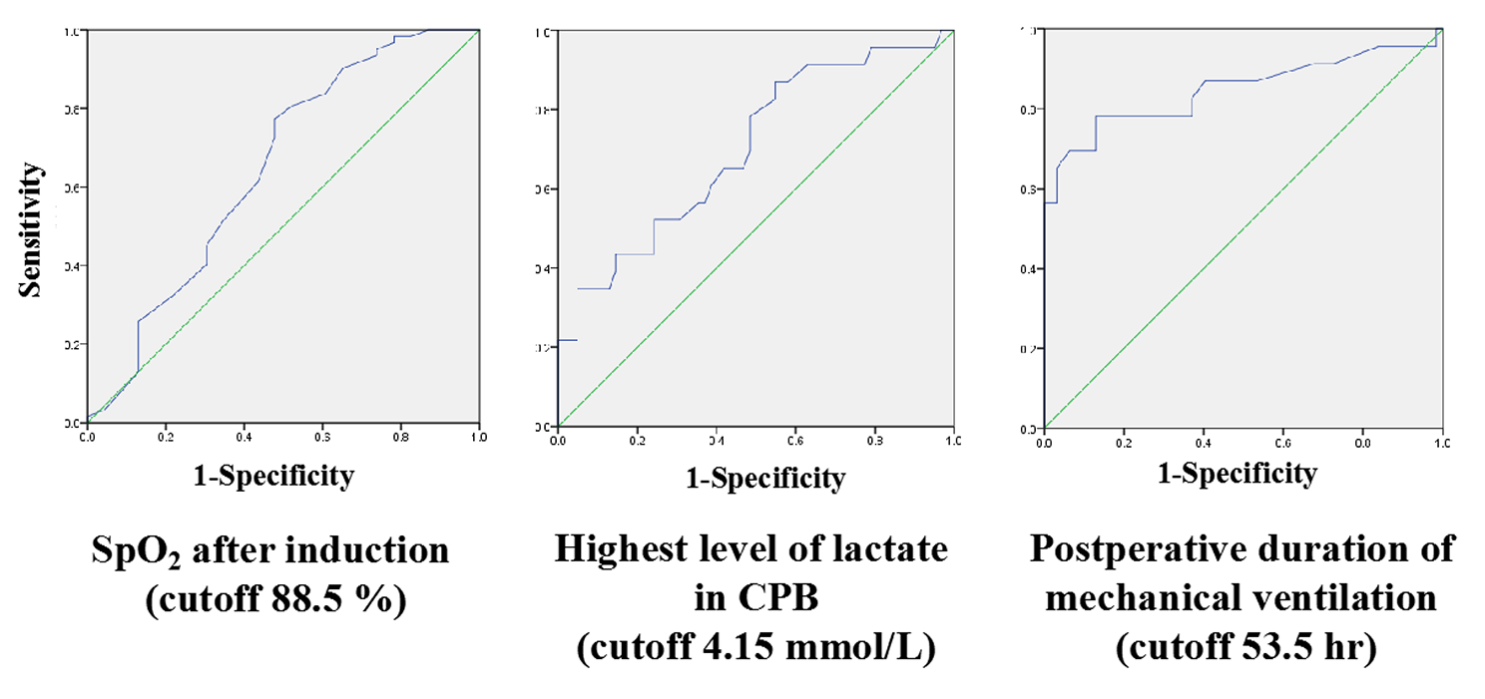
ROC curves of independent risk factors for prolonged ICU stay

Table 4 shows multi-variate analysis of risk factors for prolonged ICU stay. Three independent risk factors were identified: SpO_2_ after induction, the highest level of lactate in CPB, and postoperative duration of mechanical ventilation. The cutoff was determined by receiver operating characteristic (ROC) curve as shown in Fig. 1.

**Table 4:** Multi-variable model analysis: independent risk factors associated with prolonged ICU stay

Variable	OR	95% CI	*P*
**SpO**_**2**_ **after induction (%)**	0.774	0.582–0.591	0.018
**Highest level of lactate in CPB (mmol/L)**	2.162	1.061–4.405	0.034
**Postoperative duration of mechanical ventilation (hours)**	1.099	1.030–1.172	0.004

OR, odds ratio; CI, confidence interval

Table 5 shows the comparison among different types of TAPVC in the prolonged ICU stay group. The proportion of supracardiac TAPVC (12/23) was highest in the prolonged ICU stay group. Children with subcardiac TAPVC had a 54.5% chance of prolonged ICU stay.

**Table 5:** Effects of TAPVC type on the prolonged ICU stay

Supracardiac (n/%)	Intracardiac (n/%)	Subcardiac (n/%)	Mixed (n/%)	*P*
12 (28.6%)	3 (12.0%)			0.116
12 (28.6%)		6 (54.5%)		0.207
12 (28.6%)			2 (28.6%)	1.000
	3 (12.0%)	6 (54.5%)		**0.012**
	3 (12.0%)		2 (28.6%)	0.296
		6 (54.5%)	2 (28.6%)	0.367

Note: n, number of cases in the prolonged ICU stay group; %,proportion of cases in the same type of group

## Discussion

Few studies have focused on the duration of ICU stay after surgical correction of TAPVC. This study demonstrated that lower preoperative SPO_2_, higher intraoperative plasma lactate levels, and prolonged postoperative mechanical ventilation were independent risk factors for prolonged ICU stay in children with TAPVC. The risk of prolonged ICU stay increased with a SPO_2_ < 88.5%, plasma lactate levels > 4.15 mmol/L (highest value), and postoperative mechanical ventilator duration > 53.5 h. Young age, low body weight, subcardiac type, need for vasoactive drug support, emergency surgery, long anesthesia time, low SPO_2_ after anesthesia induction, long CPB and aortic clamp times, high lactate level, low temperature, large volume of ultrafiltration during CPB, large amounts of chest drainage, large RBCs and plasma transfusion, and postoperative cardiac dysfunction may be associated with prolonged ICU stay.

This study demonstrated that low preoperative oxygen saturation was an independent risk factor for prolonged ICU stay, with a cutoff value of 88.5%. In TAPVC without pulmonary venous obstruction (PVO), all pulmonary and systemic blood returns to the right atrium to create a complete admixture by right-to-left atrial shunting, and oxygen saturation can be as high as 90% [8]. Low preoperative oxygen saturation may indicate PVO, particularly in children with SPO_2_ < 88.5%. TAPVC with PVO is severe with moderate or severe pulmonary arterial hypertension (PAH), right ventricular hypertrophy, and right heart dysfunction. Cyanosis is common in patients with TAPVC and PVO. This suggests that malformations of heart-impacting oxygenation may be associated with prolonged postoperative ICU stay. Echocardiographic evaluation is time-consuming. Xi et al. found that emergency surgery can reduce the duration of mechanical ventilation and hospitalization in patients with respiratory or hemodynamic instability [9].

Intraoperative CPB management has a considerable impact on postoperative prolonged ICU stay. This study suggested that lactate levels > 4.15 mmol/l are an independent risk factor for prolonged ICU stay. Furthermore, CPB time, aortic clamped time, and hypothermia may be associated with prolonged ICU stay. Ortrud Vargas Hein et al.reported that CPB time and aortic clamped time were independent risk factors for ICU time more than 3 days after cardiac surgery [10]. High lactate levels during CPB are primarily caused by insufficient perfusion. Aggressive management of blood flow, temperature, hematocrit, and mean blood pressure, should be considered to ensure sufficient oxygen delivery to maintain plasma lactate levels < 4.15 mmol/L during CPB. The blood supply to the lung is derived from a small number of bronchial arteries and the coronary arteries are without blood during CPB which determines the possibility of sufficient perfusion in the lung and heart tissue. In addition, despite the bypass delivering adequate calculated tissue perfusion for a given patient, variable inflammatory, microcirculatory, and mitochondrial responses during hypothermia and CPB are likely to contribute to elevated lactate [11]. Therefore, adequate myocardial protection, shortened CPB, and aortic clamp time may help decrease intraoperative lactate levels.

This study demonstrated that prolonged postoperative mechanical ventilation is an independent risk factor for postoperative ICU stay in children with TAPVC. The duration of mechanical ventilation is a key determinant of the postoperative length of stay (LOS) in pediatric intensive care units. Previous studies have demonstrated that prolonged mechanical ventilation is associated with longer ICU LOS in adult patients after cardiac surgery [12, 13]. Long postoperative mechanical ventilation is usually attributed to the presence of chest infection or worsened heart failure. The longer duration of postoperative mechanical ventilation in patients may be explained by two factors. First, infusion of excess fluid can worsen mechanical ventilator-related complications and aggravate pneumonia in critically ill patients. Second, the infusion of excess fluid and long-term application of cardiovascular drugs can weaken myocardial function [9].

This study suggests that early postoperative chest drainage and blood product transfusion may be associated with prolonged ICU stay. Pediatric cardiac surgery on CPB is associated with significant bleeding and blood transfusion requirements. Bleeding after pediatric cardiac surgery is generally related to a combination of factors, including immaturity of the hemostatic system, hemodilution from the CPB circuit, and excessive activation of the hemostatic system. Neonates are at a higher risk of bleeding [14]. Meanwhile, there is increasing concern regarding the risks and complications associated with homologous blood transfusion in the pediatric cardiac surgical population [14]. Therefore, the implementation of perioperative blood protection strategies, improvement of surgical techniques, shortening of CPB time to reduce bleeding, and blood product transfusion may shorten postoperative ICU stay.

This study found that the filtration volume may be associated with a prolonged ICU stay. The benefits of modified ultrafiltration (MUF) include improved pulmonary compliance and gas exchange and increased HCT and blood pressure levels. However, there has been a questionable impact on long-term benefits such as the duration of intubation or intensive care unit stay [14]. There isa lack of studies on ultrafiltration volume targets to achieve a net-zero or negative operative fluid balance or targeted HCT; therefore, the outcomes of ultrafiltration studies show a wide range of filtration volumes [15]. No previous study has shown a relationship between filtration volume and increased risk of operative recovery. A possible explanation is that small coagulation factors such as thrombin (39 kDa), factor IX (55 kDa), and factor X (38 kDa) may be susceptible to depletion during ultrafiltration, thereby increasing bleeding after surgery.

Our results demonstrate that early hemodynamic variables, such as VIS and MAP,may be associated with LOS in the ICU. Early hemodynamic variables such as systolic arterial pressure, diastolic arterial pressure, and serum lactate level have been shown to be strong predictive markers of LOS in the ICU after the Norwood procedure [16]. In TAPVC there is significant right atrial and right ventricular volume overload, the left atrium is frequently small, and the left ventricle is compressed by the dilated right ventricle [8]. These pathophysiological features contribute to the development of post-operative cardiac dysfunction. Improved clinical management, including early diagnosis and timely surgical intervention to reduce hypoxia time and protect intraoperative cardiac function, can help reduce the ICU stay time.

This study has several limitations. First, this study was based on a single center, local practice patterns, and a small number of cases, which may limit the application of the current results to other institutions. Second, this was a retrospective study, which may have introduced a potential classification bias, and thus was subject to variations in physician practice with regard to the administration of parameters for extubation and discharge from the ICU. However, it is likely that this variability was present randomly across the cohort, with minimal impact on the final analysis. Finally, the sample size of this study was small, but there were many variables that may have affected the representativeness of the results.

## Conclusions

Taken together, lower preoperative SPO_2_, higher intraoperative plasma lactate levels, and prolonged postoperative mechanical ventilation were independent risk factors for prolonged ICU stay in children with TAPVC. SPO_2_ < 88.5%, highest plasma lactate value > 4.15mmol/L, and postoperative mechanical ventilation duration > 53.5 h, increased the risk of prolonged ICU stay. CPB time and aortic clamp time, prolonged postoperative mechanical ventilation, large volume of ultrafiltration during CPB, large amount of chest drainage, large red RBCs and plasma transfusion, and postoperative cardiac dysfunction may be associated with prolonged ICU stay.

## Data Availability

The datasets generated and/or analyzed during the current study are not publicly available but are available from the corresponding author on reasonable request.

## Abbreviations

ICU: Intensive care unit
TAPVC: Total anomalous pulmonary venous connection
SpO2: Saturation of pulse oximetry
CPB: Cardiopulmonary bypass
RBCs: Red blood cells
CHD: Congenital heart disease
ASO: Arterial switch operation
SICU: Surgical intensive care unit
VIS: Vasoactive index score
MAP: Mean arterial pressure
HCT: Hematocrit
IVS: Interventricular septum
CK-MB: Creatine kinase MB isoenzyme
ECMO: Extracorporeal membrane oxygenation
ABP: Arterial blood pressure
ACT: Activated clotting time
SPSS: Statistical Package for the Social Sciences
ACC: Aortic cross-clamp
NPT: Nasopharyngeal temperature
UF: Ultrafiltration
FFP: Fresh-frozen plasma
pH: Potential of hydrogen
POD: Postoperative day
LVEF: Left ventricular ejection fraction
ALT: Alanine aminotransferase
TBIL: Total bilirubin
DBIL: Direct bilirubin
PT: Prothrombin time
APTT: Activated partial thromboplastin time
INR: International normalized ratio
ROC: Receiver operating characteristic
PVO: Pulmonary venous obstruction
PAH: Pulmonary arterial hypertension
LOS: Length of stay
MUF: Modified ultrafiltration
